# Development of the MapMe intervention body image scales of known weight status for 4–5 and 10–11 year old children

**DOI:** 10.1093/pubmed/fdx129

**Published:** 2017-11-28

**Authors:** A R Jones, M J Tovée, L R Cutler, K N Parkinson, L J Ells, V Araujo-Soares, M S Pearce, K D Mann, D Scott, J M Harris, A J Adamson

**Affiliations:** 1 Institute of Health & Society, Human Nutrition Research Centre, Newcastle University, Newcastle upon Tyne, UK; 2 School of Psychology, University of Lincoln, Lincoln, UK; 3 Institute of Health & Society, Newcastle University, Newcastle upon Tyne, UK; 4 School of Health and Social Care, Health and Social Care Institute, Teesside University, Middlesbrough, UK; 5 Department of Public Health and Wellbeing, Northumbria University, Newcastle upon Tyne, UK; 6 School of Psychology and Neuroscience. University of St Andrews, Fife, UK

**Keywords:** children, methods, obesity

## Abstract

**Background:**

Parents tend to visually assess children to determine their weight status and typically underestimate child body size. A visual tool may aid parents to more accurately assess child weight status and so support strategies to reduce childhood overweight. Body image scales (BIS) are visual images of people ranging from underweight to overweight but none exist for children based on UK criteria. Our aim was to develop sex- and age-specific BIS for children, based on British growth reference (UK90) criteria.

**Methods:**

BIS were developed using 3D surface body scans of children, their associated weight status using UK90 criteria from height and weight measurements, and qualitative work with parents and health professionals.

**Results:**

Height, weight and 3D body scans were collected (211: 4–5 years; 177: 10–11 years). Overall, 12 qualitative sessions were held with 37 participants. Four BIS (4–5-year-old girls and boys, 10–11-year-old girls and boys) were developed.

**Conclusions:**

This study has created the first sex- and age-specific BIS, based on UK90 criteria. The BIS have potential for use in child overweight prevention and management strategies, and in future research. This study also provides a protocol for the development of further BIS appropriate to other age groups and ethnicities.

## Introduction

Evidence indicates that parental recognition of childhood overweight is limited.^1–4^ This may be due to parents using visual assessments of children and comparisons with others,^5–7^ rather than using objective measures such as body mass index (BMI) or growth charts,^6,7^ when determining child weight status. Parental ability to correctly identify childhood overweight in the future may become even more problematic because with increasing levels of childhood overweight at a societal level comes a shift in what constitutes ‘normal’ weight toward heavier weight categories.^8^ Identifying methods for improving parental recognition of childhood overweight are therefore urgently needed because, without recognition, parents are unlikely to make appropriate lifestyle changes or seek support.^2^ Research suggests that non-growth chart-based approaches should be considered^5^ and, given the methods by which parents determine child weight status and their sensitivity to its more visible manifestations,^9^ the use of a visual tool to improve recognition warrants further investigation.^7^

Visual representations of different child weight statuses have previously been developed and used for research purposes.^10–17^ None, however, are suitable for use with parents in an English setting since they do not correspond to the British 1990 growth reference (UK90) cut points for child weight status^18^ which are used nationally by the National Child Measurement Programme (NCMP) to monitor childhood overweight and obesity and inform parents of their child’s weight status.^19^ Body image scales (BIS) based on UK child populations and weight status criteria are therefore required if the utility of a visual tool in improving recognition of childhood obesity by English parents is to be examined. Since 4–5 and 10–11 year old children are those age groups monitored nationally,^19^ the aim of this study was to create sex- and age-specific BIS of known weight status for children aged 4–5 and 10–11 years using UK90 criteria.^18^

## Methods

### Participants and demographic data

Children aged 4–5 and 10–11 years were recruited at public events and venues in Newcastle upon Tyne, England, local primary schools and weight management groups. Child age and sex were recorded. Ethnicity data were not collected on the children or their families and pubertal stage data were not collected on the children. Parents/guardians provided written consent for their child’s participation. 10–11 year old children provided written assent.

### Measures

#### Anthropometric measures

Height was measured to 0.1 cm using a Leicester portable height measure (Chasmors, London, UK) with the head in the Frankfort plane. Weight was measured to 0.1 kg in light indoor clothing using Tanita scales. Measurements were taken until two values were obtained within 1.0 cm of each other for height and within 0.1 kg of each other for weight. The mean for each measure was used.

#### 3D surface body scans

A precise representation of each child’s body size and shape in 3D (http://www.ncl.ac.uk/hnrc/research/project/4348) was captured from 3D surface body scans using a mobile KX-16 3D body scanner ([TC]^2^ Labs, Apex, NC, USA). Children stood in light-coloured underwear and adopted a standard pose for ten seconds within a private cubicle whilst white light was projected onto their body. The scanner contained a set of 14 infra-red depth sensors arranged around the body, each individually fixed to the frame of the cubicle. Based on the reflected light, a large number of points in 3D space were calculated which corresponded to the size and shape of the child’s body. The scan data were stored off-line by the scanner software, and converted into a polygon mesh.

### Procedure

Height, weight and up to three body scans were obtained. Parents were invited to register their interest in the follow up qualitative stage of the study to inform the development of the BIS. Participants involved in the qualitative phase were recruited from those parents who registered an interest, Voice North (the north east research and engagement panel based at Newcastle University) and team contacts with health professionals working in the field of childhood obesity.

### The development of the BIS

#### Quantitative aspect

**Fig. 1:**
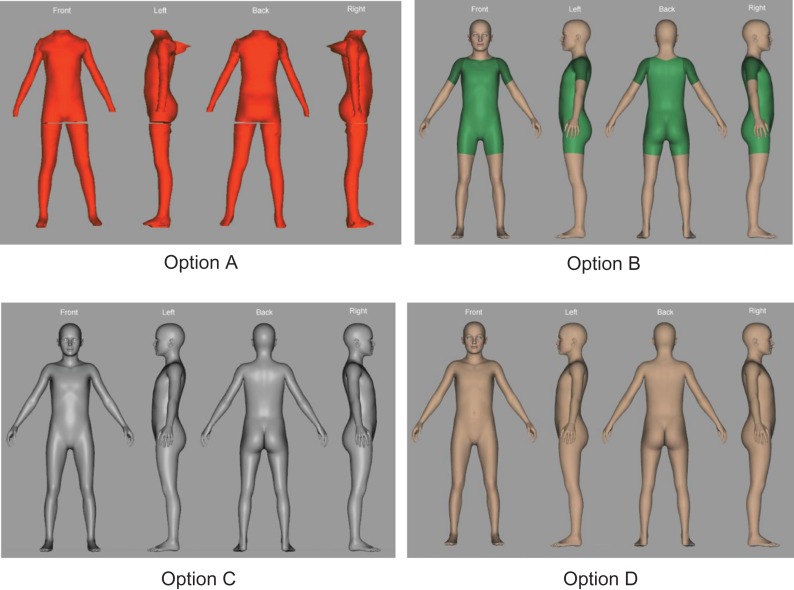
Prototype images used during qualitative discussion sessions.

Child height and weight measurements were used to calculate BMI (weight (kg)/height (m^2^)), and child weight status using UK90 criteria.^18^ Thus the weight status of each child *and* their associated 3D body scan was known. To create a BIS, for each age and gender, the 3D body shapes for all children within a particular weight category were averaged and a single 3D representation of the size and shape of all the constituent bodies in that category was produced. The seven weight categories used were: underweight (≤second centile, clinically low weight); lower-healthy weight (2.1–49.9th centile, clinically healthy weight); mid-healthy weight (50.0–74.9th centile, clinically healthy weight); upper-healthy weight (75.0–90.9th centile, clinically healthy weight); overweight (91.0–97.9th centile, clinically overweight), lower-very overweight (98.0–99.5th centile, clinically obese) and upper-very overweight (≥99.6th centile, clinically extremely obese). The weight categories represented were guided by the centile cut-offs used by the NCMP^19^ and their labelling terminology, i.e. ‘very overweight’ was used instead of ‘obese’. A variety of ‘skins’ were mapped onto one of the averaged shapes to produce a range of prototype images for use in the qualitative stage of work described below (Fig. 1). Following the qualitative work and team discussions it was deemed that although the 3D physical dimensions of the prototypes were anthropometrically accurate, none looked sufficiently realistic. A more realistic representation of the bodies was therefore needed and so a 3D modelling software package (Daz Studio 4.5 from www.daz3d.com) was used to create photorealistic 3D models. The advantages of this method are that the same identity of the body in the image for each image set is clearly maintained over a wide BMI range and the 3D rendered stimulus images are high definition and photorealistic.^20^ The female and male 3D models used were Victoria 5.1 (V5) and Michael 5.0 (M5), respectively. The program has been used previously to create sets of bodies for adults and children^21–23^ which were rated for characteristics such as health and body weight in the same way as digital photographs of real bodies were rated.^24^

The bodies used to create the BIS for girls were shaped using the V5 morphs, the Genesis Evolution head and body morphs (including the Genesis Child morphs), the feminine touch morphs (all from www.daz3d.com) and the preteen morphs (from www.renderosity.com). These morphs allowed the artificial bodies to be altered in over 100 independent body shape dimensions, providing fine control over the resultant size and shape. The bodies were then covered using the ‘Pippa’ skin textures and hair (www.daz3d.com). The bodies used to create the BIS for boys were shaped using the M5 morphs, the Genesis Evolution head and body morphs and the preteen morphs. The bodies were covered using the elite ‘Jeremy’ high-resolution skin and the ‘Duke’ hair package (both from www.daz3d.com). Bodies for both genders were clothed in the Genesis Tankini and Briefs.

To create each individual body, we first imported the appropriate average scanned body into Daz Studio 4.5, and next to it the appropriate Daz 3D body model was opened. The two bodies were compared and Daz morphing tools used to restructure the Daz base model to have the same shape as the averaged body, and the same height and body proportions. When the modelling was completed, Daz Measurement Metrics (v1.1) tools were used to measure the following circumferences from the Daz model: bust, under-bust, waist and hips and compare them to the equivalent measures from the scanned data provided by the [TC]^2^ scanner software. The criteria for an adequate model fit to the scan data were that the key measurements from the Measurement Metrics were within ±5% of the scan data; there was minimal distance between the averaged scan body surface and the model surface throughout the entire model and that there was a good qualitative fit between the scanned body and the Daz model (i.e. the Daz body should obviously look like the scanned body).

#### Qualitative aspect

Discussion sessions were conducted with parents and health professionals working in the field of childhood obesity to explore and inform the development of the BIS. To maximize participation in this phase of work a pragmatic approach was taken and so participants were invited to participate in either focus groups or individual interviews depending on their personal preference and availability. Mothers and fathers were invited to participate in parent sessions which were conducted separately to those involving health professionals. Written consent was obtained from all participants and confidentiality in reporting was assured. Sessions followed a semi-structured guide designed to encourage group interaction and discussion with the interviewer; this included an examination of the prototype images (Fig. 1) and incorporated questions such as ‘What are your views on the images developed?’, ‘How could they be improved?’ and ‘What do you think we need to change?’. Discussion sessions were audio recorded and transcripts reviewed for accuracy by two independent study team members.

Data analysis was conducted (A.R.J.) using NVivo 10 (QSR International Pty. Ltd., Doncaster, VA, Australia). Focus group and interview data were assessed collectively and the discussion guide headings directed the analysis process that involved the systematic and continuous development of categorization codes that organized the data and identified important topics and issues.^25^

Quotes are used for illustrative purposes and are tagged by participant type (Parent, Health Professional), session type (Focus Group, Interview) and number (1–5 for focus groups, 1–6 for interviews), and identity number (ID, for focus groups).

A favourable ethical opinion for the study was obtained from the Faculty of Medical Sciences ethics committee, Newcastle University and Newcastle and North Tyneside 2 National Research Ethics Service Committee.

## Results

Parental consent was obtained for 598 children (396 (66.2%) 4–5 year old, 50.8% boys; 202 (33.8%) 10–11 year old, 47.5% boys). Most of the north east of England population (95%) describe their ethnicity as ‘White’^26^ and so those children recruited were predominantly Caucasian. Following recruitment some children, particularly in the younger age group, became less confident about completing the scanning procedure and/or moved excessively during the process thus rendering the data collected unsuitable for inclusion in analysis. Complete data (height, weight and useable 3D scan data) were therefore obtained from 388 children (211: 4–5 year old, 53% boys, 7.6% overweight or very overweight; 177: 10–11 year old, 45% boys, 26.0% overweight or very overweight). Using the complete data, the prototype images shown in Fig. 1 were produced for discussion in the qualitative aspect of the study.

### Qualitative aspect

Five focus groups and six interviews were completed with parents (27 mothers and 6 fathers), one focus group was completed with four health professionals working in the field of childhood obesity.

When discussing the prototypes participants preferred option B due to its more ‘human like’ nature and the ability to relate the images to their own child (Table 1). Some parents also preferred the images being clothed. Option A was least favoured because of its ‘depersonalised’ nature and lack of definition. Option C was generally not favoured due to its unrealistic nature and lack of contrast to the background. Table 1 shows views on option D were mixed, some stated they could relate such images to their child and that they were ‘natural looking’. Some, however, felt this option was not realistic, they had no definition and contrast, and they preferred the clothed images.

**Table 1:** Participants’ views on the prototype body image scales

Prototype	Quotes
A 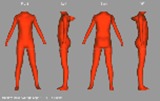	‘I kinda [of] feel like option one [A] and option three [C] really aren’t, as far as I’m concerned really suitable…I think it’s important to have… heads, faces to make it easier…For parents to understand, so option one without the heads I think… it’s not as well defined I guess’ [Health Professional, FG1, ID3] ‘I think again it is just that the lack of, erm, feeling that it’s somebody…I think it is just the lack of erm, I wouldn’t relate it to my child I would just think it looks more like a superman suit or something’ [Parent, INT1] ‘This one you feel that it’s just completely… disembodied and not a person at all…They haven’t got their heads on’ [Parent, INT2]
B 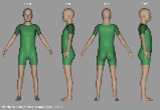	‘I like the style of number two [B] because its…to me I can see it more easily as a human body for me number two…it’s nice to have it differentiated with the skin and then like a body suit on at that level. I like seeing the body suit cos [because] it gives me an idea of what a child would look like I see that clearly’ [Parent, FG1, ID5] ‘a real child looks like option 2 [B]…with the swimsuit on and that’s often you know if you [are] in the [swimming] baths you see kids running round with these swimsuits on and that’s more like you’d see a child in everyday life’ [Parent, FG3, ID5] ‘I think that’s hit the nail on the head really I think number two [B] is going to be more user friendly because it is more like a child’ [Parent, FG3, ID3] ‘And this one feels a little bit more human. I think it’s because he’s got clothes on, he or she, it could be androgynous but he’s got clothes on and…you can get a feel for it being a real person’ [Parent, INT2]
C 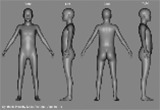	‘This one looks a bit like…mechanical…like…terminator style’ [Parent, INT3] ‘…sort of playing a trick on me [my] eyes because if they’re the same size I would say number three [C] looks slimmer…It’s really looks slimmer…Three [C] looks slimmer than four [D]…It’s a tone thing it’s just looking toned’ [Parent, FG1, ID5] ‘The silver ones a bit too reflective, it looks a bit weird like they’re all sort of wet and shiny’ [Parent, FG3, ID1]
D 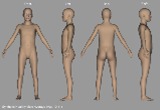	‘I don’t like this one…that didn’t look like a naked child’ [Parent, FG2, ID2] ‘So I think these two [B and D]…But then you see because they’ve got no clothes on, people will say put some clothes on’ [Parent, INT3] ‘…because it’s like actually looking at a person’s body. It’s like more natural sort of thing, it’s like what you would like actually see in your own child’ [Parent, INT4] ‘…whereas I automatically took 4 [D] and 2 [B] when I thought of [child’s name] so I think, you know, initially I’m like oh that looks like [child’s name], you know, and I think to make me think to relate it to my child I need to be able to see that it’s got a head and that it’s got skin colour’ [Parent, INT1]

FG, focus group; INT, Interview.

Other issues and topics discussed during the discussions are shown in Supplementary Table S1. Realism was particularly important for participants, the importance of enabling parents to relate the BIS to their child/children in general was highlighted as was presenting images which had age appropriate faces and body proportions. The use of colour on the BIS and adding hair to the images were also commonly discussed issues. The associated meanings of colours was highlighted as an important consideration as were clarity and not detracting from the purpose of the scales. In most instances the addition of hair to the images was not deemed necessary or there was no preference on this matter. Some participants felt though that adding hair would provide more realistic images, and be useful to differentiate between boys and girls. In both instances participants discussed the importance of enabling parental use of the scales and not personalizing them to a degree which would impact on parents relating them to their own child (Supplementary Table S1).

The potential impact of the images’ Caucasian skin tone on their perceived relevance to black, minority ethnic (BME) communities was discussed during some sessions. Participants were informed that the current BIS were not ethnic specific but it was evident that developing inclusive and ethnic specific scales was important. Other suggested improvements included altering the stance of the images, adding the BMI centiles represented in each image or stating the BIS have been created in line with NCMP criteria, and considering the pubertal stage of the children.

When discussing the usefulness and acceptability of the scales, participants in most sessions stated that they would be useful or that other parents would find them useful and acceptable (Supplementary Table S1). Health professionals also indicated that the BIS could be helpful for those working in the field of childhood obesity but that it was important to use terminology consistent with that used by the NCMP (i.e. very overweight rather than obese) on the BIS for acceptability and consistency purposes. Methods of presenting and promoting the BIS to parents (e.g. via schools, media campaigns) were also discussed but some participants expressed concern that the BIS would be questioned, that they may not be used by parents who perceived childhood overweight to be irrelevant to them and in some instances parental action may only take place following discussion with others such as a family member or health professional.

Participants also discussed which viewpoints of the images would be of most use to parents. There were mixed views on this but participants in most sessions stated they preferred the images being presented from three angles (front, back, and one side angle). Presenting two angles (front and one side angle) was also commonly chosen by participants, as was all four (front, back, both sides), however, it was acknowledged by some that presenting the images from all viewpoints in paper format may be difficult.

### The MapMe BIS

**Fig. 2:**
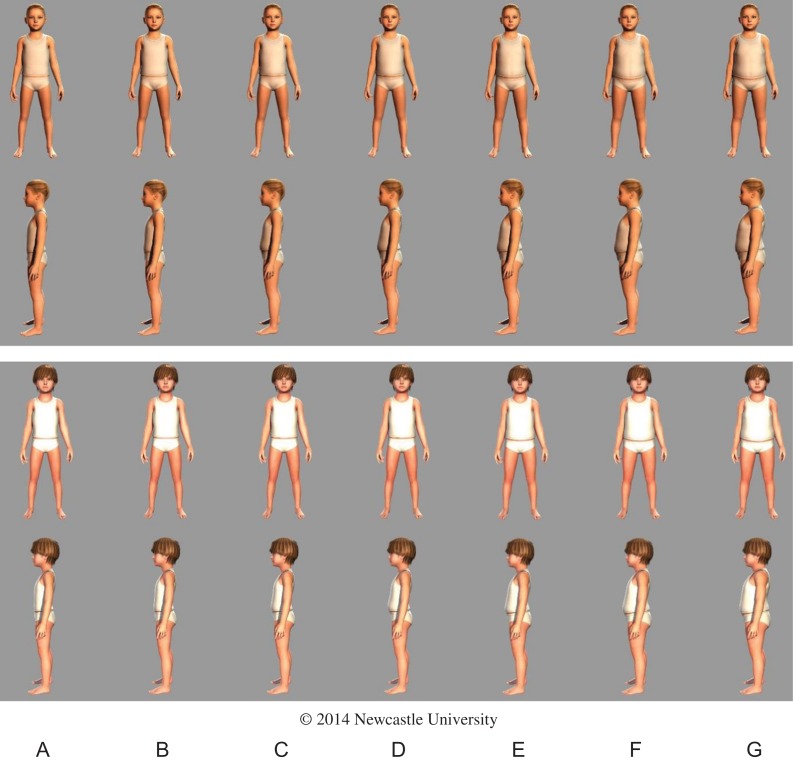
Body image scales of known weight status for 4–5-year-old girls and boys (A = underweight; B, C, D = healthy weight; E = overweight; F, G = very overweight). Readers who wish to use this image should contact Prof Ashley Adamson (Ashley.Adamson@newcastle.ac.uk), for detailed instructions on how to use and insert the image into other documents.

**Fig. 3:**
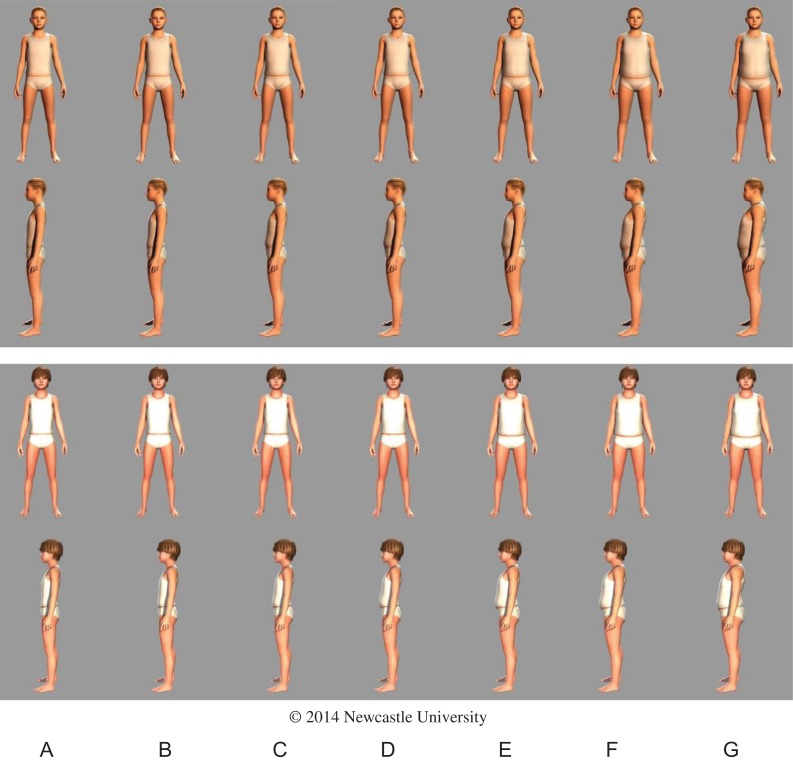
Body image scales of known weight status for 10–11-year-old girls and boys (A = underweight; B, C, D = healthy weight; E = overweight; F, G = very overweight). Readers who wish to use this image should contact Prof Ashley Adamson (Ashley.Adamson@newcastle.ac.uk), for detailed instructions on how to use and insert the image into other documents.

Using the quantitative and qualitative data, BIS of known weight status using UK90 criteria^18^ for 4–5 and 10–11 year old were developed (Figs 2 and 3). Table 2 describes the weight categories of each image and the range of BMI centiles represented in each image. In a small number of cases useable data were unavailable, e.g. for upper-very overweight girls aged 4–5 years, so data for boys in this weight and age range were used. In these young children, for a given age and weight category, the body shapes for boys and girls were extremely similar (Figs 2 and 3), and so we judged that the equivalent body of the other gender represented a good approximation of the body size and shape of any missing bodies. The BIS were created in paper-based and web-based format. Front and side viewpoints were presented in the paper-based format. In the web-based format each image can be seen in 3D and rotated 360° to enable examination from all aspects.

**Table 2:** Descriptive data for each body image scale

		Image						
		A	B	C	D	E	F	G
	BMI centile cut-points for image	≤2.0	2.1–49.9	50.0–74.9	75.0–90.9	91.0–97.9	98.0–99.5	≥99.6
	Weight category (NCMP^a^ terminology)	Underweight	Healthy weight	Healthy weight	Healthy weight	Overweight	Very overweight	Very overweight
	Weight category (clinical definition)	Underweight	Healthy weight	Healthy weight	Healthy weight	Overweight	Obese	Extremely obese
Boys: 4–5 years	Number of scans	Girls data used	44	42	16	7	1	1
	Range of BMI^b^ centiles represented	Girls data used	4.27–49.78	50.02–74.37	77.74–88.66	91.52–97.20	99.21	99.74
Girls: 4–5 years	Number of scans	3	31	40	19	6	1	Boys data used
	Range of BMI^b^ centiles represented	0.22–1.96	12.92–49.98	50.52–74.97	75.26–89.39	95.02–97.75	98.77	Boys data used
Boys: 10–11 years	Number of scans	Girls data used	27	18	15	8	12	Girls data used
	Range of BMI^b^ centiles represented	Girls data used	3.31–48.65	56.59–74.53	77.01–90.88	91.96–97.06	98.27–99.48	Girls data used
Girls: 10–11 years	Number of scans	1	34	18	18	15	8	3
	Range of BMI^b^ centiles represented	1.42	3.83–49.11	50.65–74.28	76.84–90.47	91.29–96.86	98.19–99.45	99.94–99.92

^a^National Child Measurement Programme.

^b^Body mass index.

## Discussion

### Main finding of this study

With involvement of parents and health professionals working in the field of childhood obesity this study has created the first BIS of known weight status for 4–5 and 10–11 year old based on UK90 criteria.^18^

### What is already known on this topic?

Visual representations of children of different weight statuses have been created previously.^10–17^ None though mapped on to the UK90 cut-points used by the NCMP in England, and have therefore been inappropriate for use with families with young children in England. The methodologies used to create previous visual tools have also varied. For example, silhouettes were developed using children’s body measurements and evaluations by medical experts in the field of childhood obesity for accuracy in their representation of different weight categories^17^ whereas the toddler silhouette scales described by Hager *et al.*^11^ were produced by providing an artist with 15 photographs of toddlers with their weight-for-length percentiles and caregivers’ perceptions of toddler features associated with different weight statuses. Reifsnider *et al.*^14^ also attempted to create child silhouettes but they were unrealistic because they resembled adult body proportions and so three photographs were used. Eckstein *et al.*^10^ describe how digital images of children across age and weight categories were viewed by a graphic artist to create sketches which were modified following discussion with team members experienced in child nutritional assessment. The middle image in each series of sketches was created to represent a child on the 50th percentile, other sketches were not attached to any particular BMI percentile category. Huang *et al.*^12^ also used photographs (12, spanning four age groups) which presented front and side views of the children, and the BMI-for-age and sex percentile was known for each child. The Children’s Body Image Scale^16^ was again created using photographs of children of known BMI. However, each image was created to represent the range of BMI from the mid-point of the image below and above.

### What this study adds

Our study recruited a large group of children and utilized 3D surface body scanning technology in conjunction with objectively measured weight status to create the BIS. The images presented are averages of those children within each weight category and so provide a more accurate representation of each weight status. The BIS were also created with extensive input from parents and health professionals who work with families to maximize their acceptability.

The BIS developed in this study have important implications for both the scientific community and clinical practice. They have been created in two formats and have been tested, as part of an intervention (MapMe), to examine their impact on parental recognition of childhood overweight and child weight status 12 months post-intervention.^27^ The BIS could be utilized in further childhood obesity related research to obtain data from families and health professionals on their knowledge, perceptions and estimates of child body size which can be corresponded to a known weight category and/or BMI range. The BIS could be added to national epidemiological surveys as a proxy for weight status alongside reports of height and weight. Researchers working in other fields, such as body image, could also use the BIS which could be examined by children themselves in order to explore self-image and auto-evaluation of body image. The BIS may also be of benefit to personnel working with families, in starting conversations about the issue of childhood overweight and monitoring progress of healthy weight maintenance efforts. The BIS have potential nationally; they could be used to support the NCMP process of informing parents of their child’s weight status as parents are often shocked and in disbelief following receipt of this information.^28^ Finally, the BIS could also be utilized to inform caregivers and health professionals about childhood obesity and tackle the issue of increased threshold for perception of overweight.^8^

### Limitations of this study

Limitations include the difficulties in obtaining analysable 3D surface body scan data from children (e.g. due to excessive movement of the child during the scanning process). In addition, children recruited were predominantly Caucasian and so the utility of the BIS with BME communities is unknown; further testing and BIS development is therefore needed prior to use with BME families. Despite active recruitment from public venues, primary schools and weight management groups locally, the number of overweight/obese children from which data were used for the BIS development was lower than those seen in the healthy weight categories meaning less variability in body shape and size being represented for these groups. For some unhealthy weight categories no useable data were available, this further highlights the difficulties and sensitivity of involving families with unhealthy weight children in this type of research. Future work could therefore further recruit unhealthy weight children in order to enhance and extend the existing BIS. Finally, reliability and validity testing of the BIS developed was not within the scope of this study but these important concepts are the subject of on-going work.

## Conclusion

In conclusion, we have created BIS of known weight status for children aged 4–5 and 10–11 years using UK90 criteria. These new BIS have huge potential for public and clinical health practice, and future childhood obesity related research. They are available in two formats to increase their utility in different settings. Further work is needed to evaluate the use of these BIS with BME families.

## Supplementary Material

Supplementary Data

Supplementary Data
