# Supplementary material for: Development of the MapMe intervention body image scales of known weight status for 4–5 and 10–11 year old children
Source: J Public Health (Oxf). 2017 Nov 28;40(3):582–90. doi: 10.1093/pubmed/fdx129 (PMC6266708; doi:10.1093/pubmed/fdx129)
Supplement: Supplementary Data [file fdx129_table1issuesandtopicsqualitativesessions_postrevisions_cleancopy.docx]

Electronic Table 1 Issues and topics discussed during qualitative sessions

| **Issue/topic** | **Quotes** |
| --- | --- |
| Realism | “I think the overall feeling was to be as human as possible wasn’t it” [Health Professional, FG1, ID4]  “..the more realistic it is…obviously the better it is to interpret if ya [you are] looking at your own child, I think” [Parent, FG2, ID1]  “…has it [the computer programme] been told eleven or twelve…it just doesn’t look any age…I think, yeah, we accept that it’s not real but [it should look] looks about that age” [Parent, FG3, ID3]  “…the realness of that one is, is nice isn’t it…You can imagine that as being your daughter or…Your son you know what a [I] mean it’s… It’s like really life” [Parent, FG4, ID3]  “And this one feels a little bit more human…you can get a feel for it being a real person…So you can maybe understand the body images a little better” [Parent, INT2] |
| Colour | “I don’t like the red and I think the colours important and you know the red thing means danger…So green means go, red means…Danger…” [Parent, FG1, ID5]  “Maybe you could do different colour suits compared to different weights you know, blue for underweight, red for overweight, green for the right weight kind of thing and that, you know…traffic lights” [Parent, FG2, ID2]  “I’ve got a girl and looking at something blue, it’s like oh, it’s not relevant to me ya [you] know” [Parent, FG5, ID1]  “I think it’s the colours, because they are quite off-putting so you are not looking, focussing on like these which are more focused on the colour…All the colours are quite bright. If it was more neutral colours I think you can focus more…But the green is too stand outish and this one you cannit [cannot] see any definition on the red one at all” [Parent, INT4]  “I suppose what you’ve gotta [got to] think of is visual impairment as well and what colours…or lack of colour is more easily read to the wider population” [Health Professional, FG1, ID2]  “I’d watch out for colour choice as well cos [because] like if someone was colour blind I don’t know how they would see that” [Parent, FG5, ID1] |
| Hair | “I was thinking…with having boys and girls… the, the picture there doesn’t have any hair. But then when you start thinking about all different hair styles…so maybe it’s best just to keep them like that cos [because] its, its, erm, less complicated that way. You can still recognise that’s a human being” [Parent, FG3, ID5]  “It’s just with having the head like that, a [I] can see the proportions of the head to the body…And I think having hair on it might change the proportions so I’d say maybe not…Leave the hair” [Parent, FG5, ID2]  “I don’t think it really matters…I mean no it doesn’t matter...Yes I prefer it with a head…but I mean if there was hair on it, it wouldn’t make me not look at it either. It’s just it’s not something that I think needs to be added to it.” [Parent, INT3]  “That can become too personalised with hair…[be]cause you’re putting in style then in and… it also means divorcing yourself from… it looking like your child, you know the more detail you put on the more you think that’s not like…Ours…. You know what I mean” [Parent, FG1, ID5]  “Maybe if you give the female one a bit of hair then” [Parent, FG4, ID1]…“Just to get a bit of differentiation…Between male and female” [Parent, FG4, ID6]…“  See I’m thinking about my son…You know if it was a parent with a girl maybe they would identify more if it had long hair or hair but because I’ve got like a boy. Sometimes [his] hairs shaved anyway so it doesn’t [matter]…Most children at that age, well at the younger age normally have got mousy hair haven’t they. Like mousy. Like shoulder length?” [Parent, INT4]  “Just do short hair on both of them…Probably just cropped doesn’t really matter what their hair is, what type of hair it is, things like that, do you know what I mean just as long as it looks sort of huminoid” [Parent, FG2, ID2] |
| Usefulness and acceptability | “I think it’s good that it’s not a weights and heights thing…I think it’s good that it’s a shape thing…I think they are great.” [Parent, INT1]  “I think it is a far simpler way of judging if your child is obese, looking at pictures…It’s not just a fact of weighing them you want to see the images as well to compare.” [Parent, INT2]  “I think we’re always looking at new tools…we used to help raise the issue…I think that’s quite interesting cos [because] that brings up the whole issue of are they acknowledging the issue of that, if they sitting in clinic are they even ready to change, they’re probably nowhere near that, that point yet so it might inform what kind of work ya [you] then do with the parent…”[Health Professional, FG1, ID2]  “…start it with perhaps through education…what was happening before the trust in that people will pick things up isn’t working so I would say that it would have to be through media and through organisations through schools through ya [you] know parent partnerships…and then recreate… a culture of, of people addressing and monitoring children’s weight and realising that it is ya [you] know, that it…is important and that that we do need to deal with it…I think that ya [you] can’t just trust people to just switch on to you accessing” [Parent, FG5, ID3]  “…I don’t know what parents reaction is likely to be, I don’t know whether they will have the same response to the charts as they do to the NCMP numbers which is, that’s wrong…I don’t know where they got those children from but it’s not, they’re no representative of whatever it is…that [is what] they say with the NCMP” [Health Professional FG1, ID4]  “I can see your scale helping people who want to do something, I’m not sure about the people who don’t want to see it because they don’t see it anyway” [Parent, FG1, ID5] |

FG, Focus Group; INT, Interview
